# A novel rodent model of spinal metastasis and spinal cord compression

**DOI:** 10.1186/1471-2202-13-137

**Published:** 2012-11-01

**Authors:** Zion Zibly, Cody D Schlaff, Ira Gordon, Jeeva Munasinghe, Kevin A Camphausen

**Affiliations:** 1 Radiation Oncology Branch, National Cancer Institute, National Institutes of Health, 10 Center Drive, Bethesda, MD, 20892, USA; 2 Surgical Neurology Branch, National Institute of Neurological Disorders and Stroke, National Institutes of Health, Bethesda, MD, 20892, USA; 3 Cell and Cancer Biology Branch, National Cancer Institute, National Institute of Neurological Disorders and Stroke, NIH, Bethesda, MD, 20892, USA

**Keywords:** Spinal metastasis, Spinal cord compression, Animal model, Rat

## Abstract

**Background:**

Spinal cord metastatic lesions affect a high number of cancer patients usually resulting in spinal cord compression syndrome. A major obstacle in the research of spinal metastatic disease is the lack of a simple reproducible animal model that mimics the natural course of the disease. In this study, we present a highly reproducible rodent model that can be used for different types of cancers while mimicking the natural course of human metastatic spinal cord compression syndrome.

**Results:**

All sixteen Fisher 344 rats survived the dorsal approach intraosseous implantation of CRL-1666 adenocarcinoma cells and both rats survived the sham control surgery. By Day 13 functional analysis via the modified Basso-Beattie-Bresnahan (BBB) locomotor rating scale showed significant decrease in motor function; median functional score was 3 for the tumor group (*p* = 0.0011). Median time to paresis was 8.7 days post-operatively. MR imaging illustrated repeated and consistent tumor formation, furthermore, onset of neurological sequale was the result of tumor formation and cord compression as confirmed by histological examination.

**Conclusions:**

Analysis of these findings demonstrates a repeatable and consistent tumor growth model for cancer spinal metastases in rats. This novel rat model requires a less intricate surgical procedure, and as a result minimizes procedure time while subsequently increasing consistency. Therefore, this model allows for the preclinical evaluation of therapeutics for spinal metastases that more closely replicates physiological findings.

## Background

A substantial number of cancer patients develop metastatic lesions in the spinal vertebra, and are associated with high rates of morbidity and mortality. Approximately 70% of patients with advanced breast or prostate cancer, and 15% of lung cancer patients develop bone metastasis [1]. Of these patients, 15 to 20% will develop a metastasis to the vertebra with a significant proportion presenting with symptoms including: pain, compression fractures, anemia or neurologic sequalae, due to spinal cord or nerve root compression. Once tumors have metastasized they are usually incurable with less than 20% of patients surviving past 5 years [1,2].

Although metastatic spinal cord compression is a major cause of morbidity and mortality, a key obstacle in the study of this disease is the lack of reliable, practical and reproducible animal models to allow for the exploration of new treatment methodologies. Several researchers have attempted to replicate the disease effects for spinal metastases; however, these models have several limitations, such as high mortality rates, the use of rare metastatic routes for cell implantation, and wide ranges for symptom onset [3-6]. Better models are needed to provide more reliable predictions of novel therapeutic agents in animals, and their subsequent efficacy in humans. This preliminary data is essential for the investigation of new therapeutic agents, and the translation of laboratory advances to human cancer care.

In this study, we demonstrate a simple and highly reproducible model for spinal intraosseous metastatic cancer using the breast adenocarcinoma cell line CRL-1666 in Fisher 344 rats. Our model replicates the natural course of human metastatic spinal cord compression syndrome. We present the methodology of intraosseous tumor implantation, study the timing of functional motor loss, and observe tumor growth patterns using magnetic resonance imaging (MRI) and histopathlogic studies.

## Results

### Functional assessment

**Figure 1:**
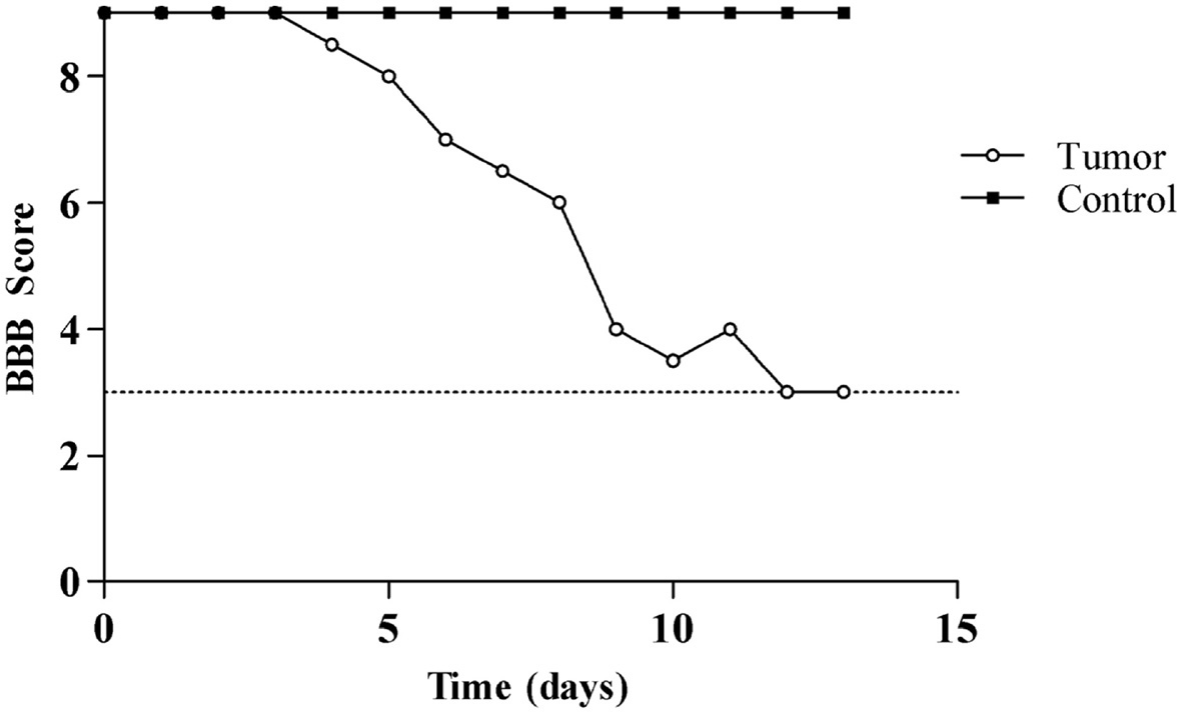
**The median BBB score of treatment group over time from date of surgery until MR imaging.** Cut-off value was a BBB score of 3.

Eighteen rats were used in this study, with all 16 receiving CRL-1666 tumor implants and 2 rats receiving sham injections. All 18 animals survived the surgical procedure and the immediate postoperative period without complication. Functional assessment was monitored using the modified BBB scale as described. The graph in Figure 1 illustrates the median modified BBB score over time. The mixed polynomial model was implemented to analyze the functional status of the rats from the date of the surgical procedure until the MR imaging. The median time to paralysis was 8.7 days. Thirteen days post-operatively the median modified BBB score was 3 for the tumor group, while the modified BBB score for the Sham group was consistently 9 (*p* = 0.0011; 95% Confidence Interval; Wilcoxon signed-rank test).

### Imaging

**Figure 2:**
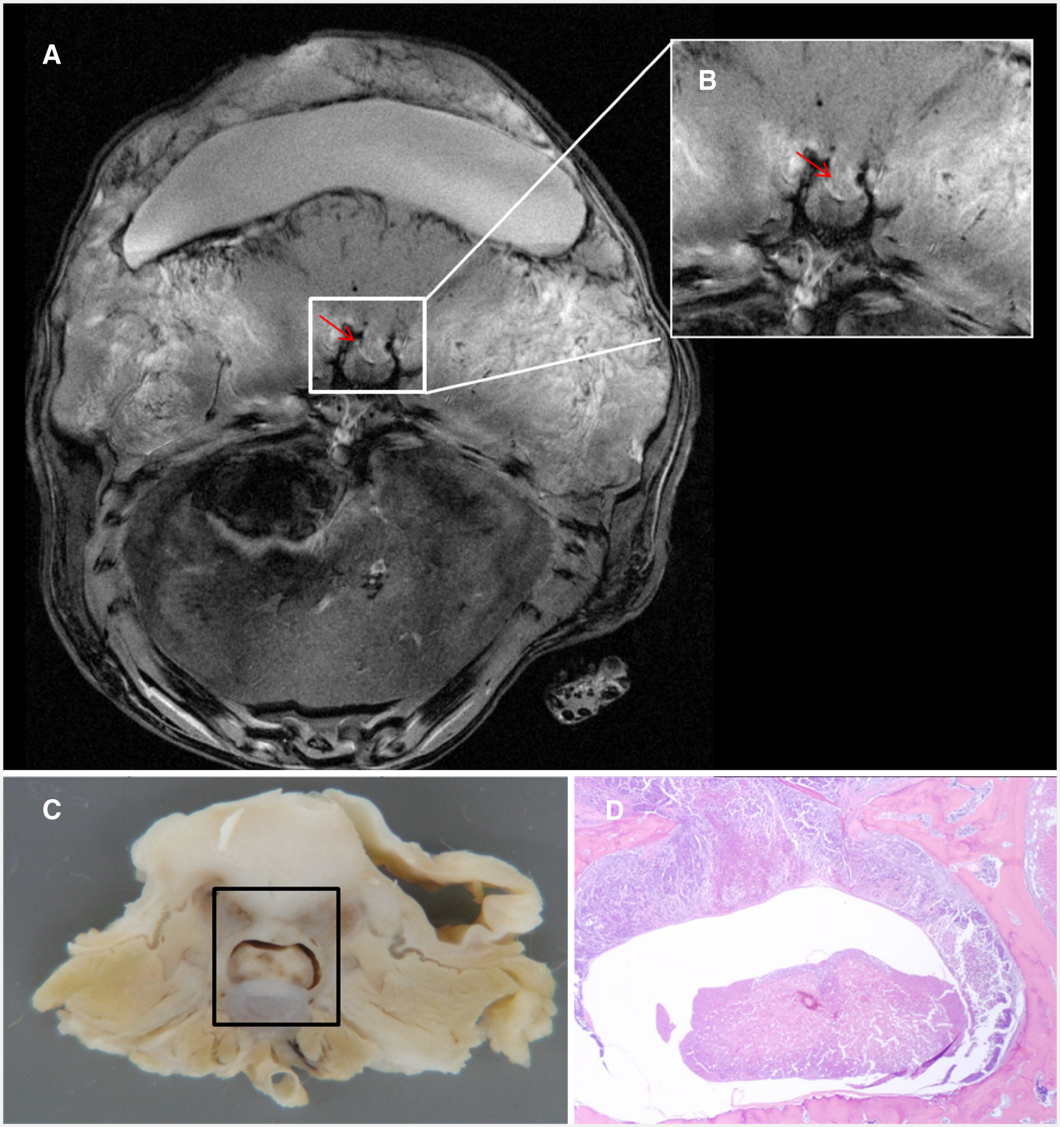
**T1 weighted post contrast MRI showing the destruction of the inner cortex of the laminae and cord compression by the tumor (red arrow in both frames).** (**A**) Higher magnification of the spinal cord and invading tumor compressing the cord (**B**) Gross anatomical section of rat spinal cord showing tumor invasion and destruction of the vertebra as well as spinal cord compression (outlined box). (**C**) Histological cross-section of rat vertebra exhibiting tumor infiltration (T; stained purple) into the spinal canal and vertebra (pink) (**D**).

As shown in Figure 2, the growth pattern of the implanted tumor was circumferential in the epidural space, and included destruction of the inner cortex of the lamina. As a result, the growing tumor mass exerted pressure on the spinal cord and evoked neurological sequalae. A part of the tumor grew posterior to the lamina with neither clinical nor neurologic consequence.

### Histopathological examination

As shown in Figure 2C and D, the gross anatomy and H & E studies of the animals that were transplanted with the CRL-1666 tumor cells, revealed a well circumscribe highly cellular lesion invading and destroying the inner cortx of the vertebral laminae. Also we could note a well area where the tumor was compressing the spinal cord and changing its shape. The tumor showed typical histopathological appearance of adenocarcinoma.

## Discussion

In this study, we describe a novel rat model for spinal metastases; describe the surgical technique for tumor implantation, in addition to, the functional, MR and histological findings. We found that rats implanted by this surgical technique with CRL-1666, a breast adenocarcinoma cell line exhibited paresis in 8.7 days, with a consistent decrease in BBB score. Animals that underwent sham surgery recovered uneventfully and maintained a maximum BBB score throughout the study.

A number of researchers have tried to establish a relevant animal model of spinal vertebral and epidural metastatic cancer. Ushio et al described a model employing paraspinal injection of tumor cells [3,4]. This model requires local invasion of the spine by tumor cells, an infrequent mode of spinal metastasis, rather than tumor growth from within the bone of the vertebra. The pathophysiology of this paraspinal injection is completely different than the typical intraosseous metastasis because it is believed that the tumor invades the vertebra from the paraspinal tissue not from the vasculature. In addition, the onset of symptoms of paraplegia required 3 to 4 weeks, restricting the efficiency of the model and its subsequent research potential.

In an attempt to address these limitations Mantha, et al demonstrated a model of spinal metastasis using a trans-abdominal vertebral implant of a breast cancer cell line [5]. Despite onset of paresis occurring within 14 to 16 days post surgery, an improvement upon the Ushio model, this model requires a highly trained surgeon who must perform a delicate and lengthy procedure to separate the great vessels from the vertebral column. Moreover, a high rate of complication, 11%, and mortality was seen. This is likely due to the complex dissection in the retro-peritoneal space required for obtaining adequate exposure to the vertebral column. Strub et al. demonstrated a metastatic renal cell carcinoma model via intra cardiac injection of tumor cells [7]. The limitations of this model were the variable time to disease and the unpredictable location of metastatic growth.

Here we described a novel, simple, and reproducible technique designed to better model spinal intraosseous metastatic cancer. Following intraosseous implantation of cancer cells in the thoraco-lumbar vertebra, image findings of epidural spinal cord compression were accompanied by neurologic findings of progressive paraparesis. The median time frame to develop paralysis was 15 to 18 days with a median value of 8.7 days; thus this model carries a high degree of consistency, illustrated by the tightly grouped decrease in functional score, and can be used as a tool for *in vivo* studies designed to evaluate the pathophysiology and treatment of spinal metastatic cancers.

One possible explanation for the observed increased rapid onset of paralysis can be the result of the chosen implantation site. Previous implantation sites used by Mantha et al. injected tumor cells into a bur in a much thicker part of the vertebrae [5]. Our bur site near the spinal process requires the tumor to grow through much less bone to reach and compress the spinal cord. The dorsal approach our group implemented compared to a ventral approach used by Mantha et al. may further help to explain this observed phenomenon [5].

The change in the neurological status of the animals, as described using the modified BBB scale, correlated well with the findings in imaging studies. The more pressure the tumor imposed on the spinal cord, as assessed by anatomical findings the more severe the neurologic deficit the animal manifested. Often the tumor was seen eliminating the subarachnoid space and inducing focal spinal cord edema. The tumor invasion and subsequent spinal cord compression confirmed by histology further validated the observed neurological change.

These findings mimic the recognized presentation and step-wise progression of spinal cord compression syndrome that is seen in humans suffering from metastatic spinal cancer [8]. This step-wise progression suggests like the model created by Mantha et al., [5] three phases of disease progression: (1) the primary or initial phase marked by similar modified BBB scores to controls; (2) the initial onset phase in which there is a slight deficits observed, and (3) the rapid progression to paralysis. There were no surgical complications, and all animals survived the procedure and developed the predicted neurological sequelae of the disease.

## Conclusions

The current model for spinal metastatic cancer involves various tumor cell lines including breast adenocarcinoma, prostate cancer, and lung cancer. This improved model is capable of consistent and reproducible tumor growth which mimics spinal cord compression and associated neurological sequale. Furthermore, the surgical procedure to implant tumor cells intraosseously is much simpler, well-tolerated, and requires less technical skill and surgical time when compared to current models. This model reliably recapitulates the human syndrome, and can be used with various tumor cell lines including breast adenocarcinoma, prostate cancer, and lung cancer. The described *in vivo* model can be capable of supporting preclinical research that advances understanding and treatment of spinal metastasis.

## Methods

### Animal and experimental design

Eighteen 10-12 week old Fischer 344 female rats weighing between 170 and 200 grams were used in this study. Sixteen underwent surgery and implantation of a CRL-1666 adenocarcinoma tumor sample and two were placed in the sham control group. (ATCC, Manassas VA) All animals were maintained in a standard two rats per cage environment, and given free access to water and food. All experimental protocols were approved by the Animal Care and Use Committee of the National Institutes of Health and performed under the ARRIVE guidelines [9].

### Instrumentation

Procedures were done under direct visualization via surgical telescopes. Surgical instruments included N0 7 forceps, No 15 scalpel blades, a mini Friedman bone rongeur, and a bipolar cautery device. A high-speed surgical drill with a 1 mm burr was used to remove the bony cortex of the lamina (Fine Science Tools, CA, USA). Muscle was closed sutured with 3-0 Vicryl suture (Ethicon, NJ, USA). Skin was closed using 3-0 silk suture (Look TM, USA).

### Tumor cell line/flank injection

The CRL-166 mammary adenocarcinoma cell line (American Type Culture Collection, Manassas, VA, USA) was used for this study. This cell line was established at the EG&G Mason Research Institute from a transplantable rat ascites tumor derived from the 13762 solid mammary adenocarcinoma. Cell cultures were grown in McCoy's 5a Medium modified with 10% fetal bovine serum (Gibco, Invitrogen). Cultures are maintained in 5% CO_2_ at 37°C and split approximately every 3 days.

A subcutaneous tumor was used as the source of the solid tumor grafts. Rats were anesthetized as described below and received a subcutaneous injection of 15x10^6^ CRL-1666 cells into the right hind limb. At day 12 following injection, animals were sacrificed, and the tumor was resected and sliced into 0.5 X 0.5 cm pieces to be suitable for intraosseous implantation.

### Anesthesia monitoring and imaging

MRI experiments were performed on a 7 Tesla (Bruker Avance, Billerica, MA, USA), 21 cm horizontal scanner. Animals were anesthetized with 1.5% Isofluorane and placed on a flat MRI compatible cushion and then mounted in a 72 mm (transmit receive) / radio frequency volume coil. The body core temperature was maintained at 37°C using a warm air blower and monitored by means of a rectal temperature probe. A pressure transducer (SA Instruments, Inc., NY, USA) was placed on the rats to monitor breathing rate (60±10 per min) under anesthesia; this respiratory signal was also used to synchronize image acquisition to minimize respiratory artifacts. Analgesia was provided by Maloxicam 1.5 mg/kg for 3 days following the surgical procedure.

MR images of three mutually perpendicular slices were acquired through the spine as scouts. The pilot images were used to obtain a series of, 0.75 mm thick, T_1_ weighted gradient echo images in two orthogonal axes, coronal (15 slices, in-plane resolution = 121 μm, Echo Time [TE]/Repetition Time [TR] = 4.5 ms/200 ms, Number of averages [NA] = 16) and sagittal (9 slices, in-plane resolution = 121 μm, TE/ TR = 4.5 ms/200 ms, NA = 16) planes encompassing the spinal cord region and the tumor. These images were used to identify the region of tumor invasion to the spinal cord and 15 axial images were acquired (slice thickness = 0.75 mm, in-plane resolution = 100 μm, TE/ TR = 4.5 ms/200 ms, NA = 16) to visualize the tumor and spinal cord.

### Surgical technique

**Figure 3:**
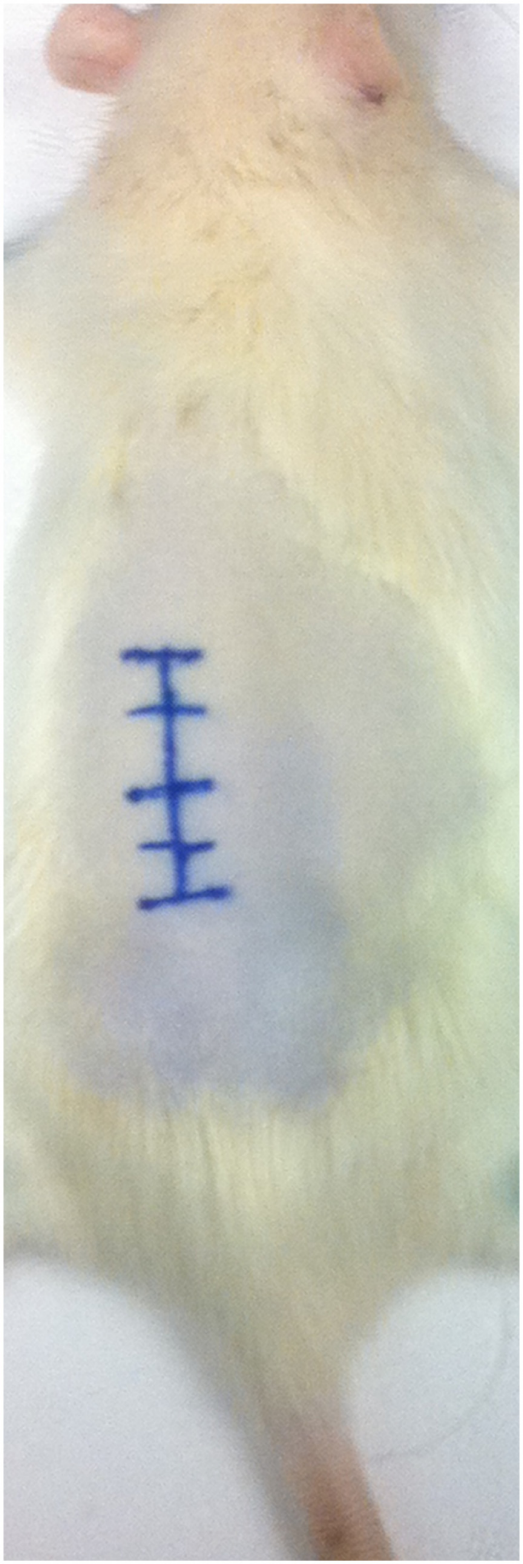
**Animal positioning and preparation prior to surgical procedure.** The thoraco-lumbar back is shaved, prepared and the mid-line skin above the spinal process is marked.

**Figure 4:**
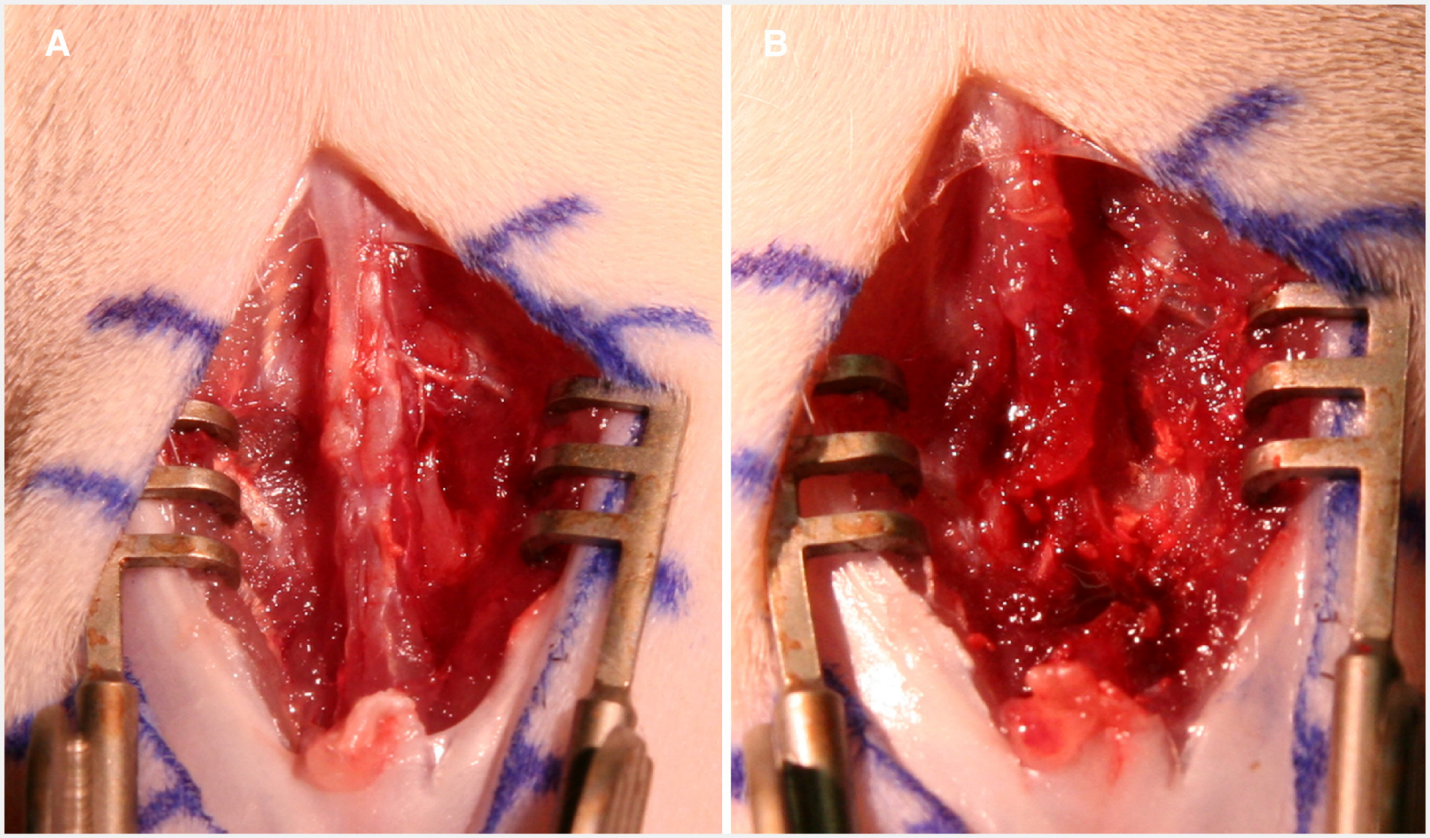
**Skin incision above the spinal process and exposing the lamina of the vertebra bilateral.** (**A**) Preparing the lamina for tumor transplant, drilling of the right lamina outer cortex after resection of the spinous process. (**B**).

**Figure 5:**
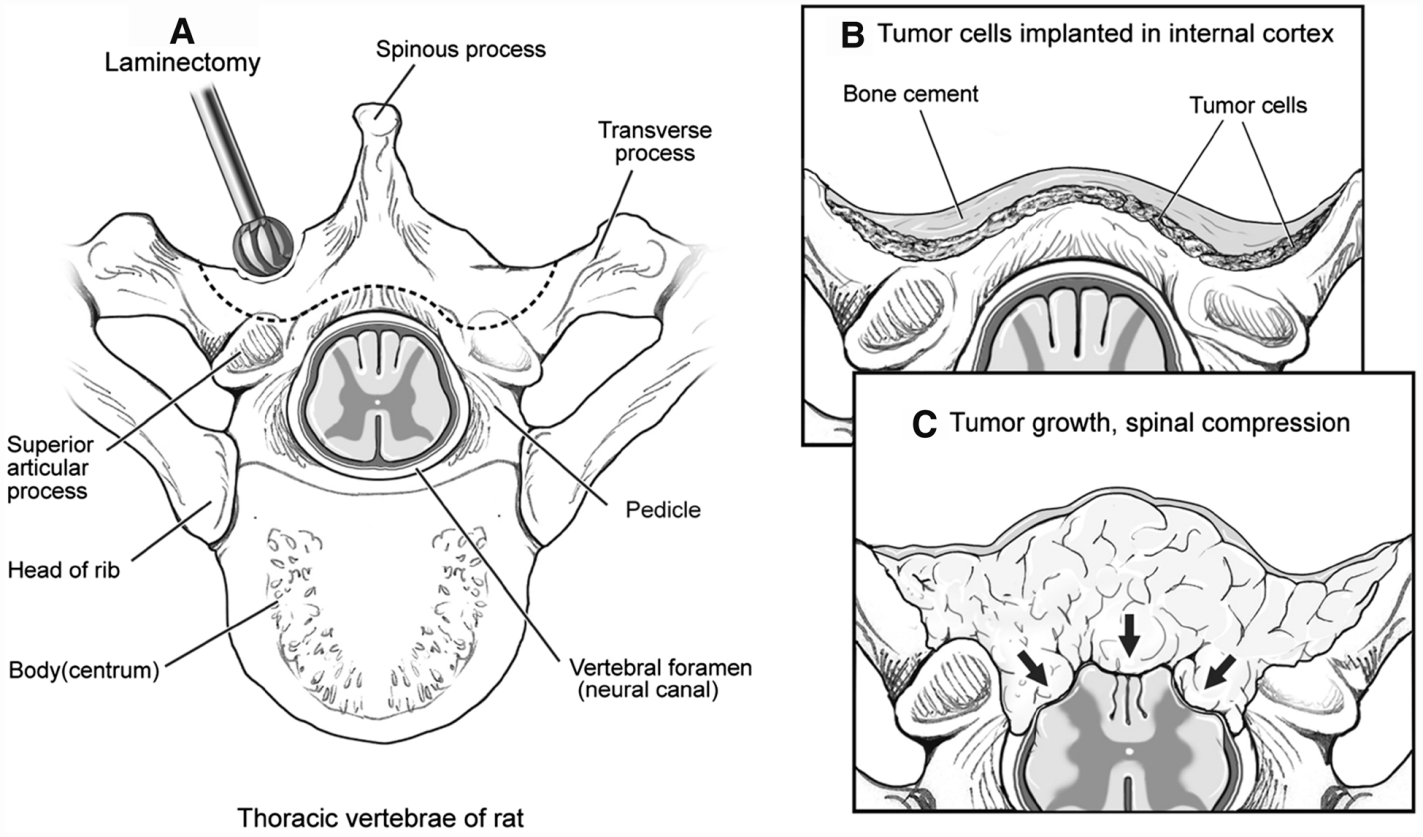
**Artist rendering of surgical procedure.** (**A**) Drilling of the outer cortex of the lamina. (**B**) Implantation of the tumor cells and covering by bone wax. (**C**) Tumor growth and spinal cord pressure.

Following anesthesia, the animal was positioned it the prone position (Figure 3). The thoraco-lumbar back was shaved and prepared with betadine and alcohol swab. A 2-cm midline skin incision above the spinal process was done and a skin retractor was used. A sub-periosteal blunt dissection of the spinalis muscle was done bilaterally to expose the lamina (Figure 4A). The spinous process was excised using the bone rounger. A high-speed drill was used to perform a partial laminectomy, drilling the outer cortex, and leaving the internal cortex of the lamina intact (Figure 4B). The cavity was 2-3 mm deep and 4-6 mm wide. The tumor piece was cut to fit the cavity size and subsequently inserted into the cavity. It was then covered with bone wax and the muscles were closed using an interrupted 3-0 Vicryl suture (Figure 5). The skin was closed with a running 3-0 nylon suture.

### Sham surgery

In the rodents undergoing sham surgery, all the aforementioned steps were taken expect the implantation of the tumor. Once the cavity was made, it was sealed with bone wax as described.

### Tissue and histopathological processing

Following animal euthanasia, the vertebral column was removed intact and fixed in 10% buffered neutral formalin. Bone was decalcified in a formic acid/sodium citrate solution. The tumor and vertebra were trimmed in cross section, embdedded in paraffin, sectioned at 5 microns and stained with hematoxylin and eosin.

### Functional assessment and statistical analysis

A single investigator assessment was done using a modified Basso-Beattie-Bresnahan (BBB) locomotor rating scale assessed daily Hind limb motor function (Table 1) [10]. Based on the animal’s gait, a score between 0 and 9 was assigned according to Table 1. Once the animal received a BBB score of 3 (paraparetic), they were anesthetized and taken to MR imaging. The raw neurological scores were summarized as the median BBB scores. The clinical significance of the change in neurological score was calculated using the Mixed Polynomial model. Column statistical analysis was done on GraphPad Prism v. 5.0.

**Table 1:** **Modified Basso**-**Beattie**-**Bresnahan rating scale**

**Score**	**Clinical symptoms**
**0**	No ankle movement
**1**	Slight ankle movement
**2**	Extensive ankle movements
**3**	Plantar placing of the paw with or without weight support -OR-
	Occasional, frequent or consistent dorsal stepping but no plantar stepping
**4**	Occasional plantar stepping
**5**	Frequent or consistent plantar stepping, no coordination -OR- Frequent or consistent plantar stepping, some coordination, paws rotated at initial contact and lift off
**6**	Frequent or consistent plantar stepping, some coordination, paws parallel at initial contact -OR- Frequent or consistent plantar stepping, mostly coordinated, paws rotated at initial contact and lift off
**7**	Frequent or consistent plantar stepping, mostly coordinated, paws parallel at initial contact and rotated at lift off –OR- Frequent or consistent plantar stepping, mostly coordinated, paws parallel at initial contact and lift off , and severe trunk instability
**8**	Frequent or consistent plantar stepping, mostly coordinated, paws parallel at initial contact and lift off , and mild trunk instability –OR- Frequent or consistent plantar stepping, mostly coordinated, paws parallel at initial contact and lift off, and normal trunk stability and tail down or up & down
**9**	Frequent or consistent plantar stepping, mostly coordinated, paws parallel at initial contact and lift off , and normal trunk stability and tail always up

## Competing interests

The authors declare that they have no competing interests.

## Author’s contributions

ZZ participated in the design of the study, experimental procedure and writing of the manuscript. CDS and IG participated in the experimental procedure and the writing of the manuscript. JM participated in the analysis of the data and the writing of the manuscript. KAC participated in the design of the study, writing of the manuscript and guidance through the project. All authors read and approved the final manuscript.

## Authors’ information

Financial support: This research was supported in part by the Intramural Research Program of the National Institutes of Health, National Cancer Institute.
